# Exogenous interleukin-6, interleukin-13, and interferon-gamma provoke pulmonary abnormality with mild edema in enterovirus 71-infected mice

**DOI:** 10.1186/1465-9921-12-147

**Published:** 2011-11-06

**Authors:** Szu-Wei Huang, Yi-Ping Lee, Yu-Ting Hung, Chun-Hung Lin, Jih-Ing Chuang, Huan-Yao Lei, Ih-Jen Su, Chun-Keung Yu

**Affiliations:** 1 Department of Microbiology and Immunology, College of Medicine, National Cheng Kung University, Tainan, Taiwan; 2 Institute of Basic Medical Sciences, College of Medicine, National Cheng Kung University, Tainan, Taiwan; 3 Department of Physiology, College of Medicine, National Cheng Kung University, Tainan, Taiwan; 4 Department of Clinical Medicine, National Cheng Kung University Medical College and Hospital, Tainan, Taiwan; 5 Center of Infectious Disease and Signaling Research, College of Medicine, National Cheng Kung University, Tainan, Taiwan; 6 National Institute of Infectious Diseases and Vaccinology, National Health Research Institutes, Miaoli County, Taiwan; 7 National Laboratory Animal Center, National Applied Research Laboratories, Taipei, Taiwan

**Keywords:** enterovirus 71, pulmonary edema, proinflammatory cytokine, mouse model

## Abstract

**Background:**

Neonatal mice developed neurological disease and pulmonary dysfunction after an infection with a mouse-adapted human Enterovirus 71 (EV71) strain MP4. However, the hallmark of severe human EV71 infection, pulmonary edema (PE), was not evident.

**Methods:**

To test whether EV71-induced PE required a proinflammatory cytokine response, exogenous pro-inflammatory cytokines were administered to EV71-infected mice during the late stage of infection.

**Results:**

After intracranial infection of EV71/MP4, 7-day-old mice developed hind-limb paralysis, pulmonary dysfunction, and emphysema. A transient increase was observed in serum IL-6, IL-10, IL-13, and IFN-γ, but not noradrenaline. At day 3 post infection, treatment with IL-6, IL-13, and IFN-γ provoked mild PE and severe emphysema that were accompanied by pulmonary dysfunction in EV71-infected, but not herpes simplex virus-1 (HSV-1)-infected control mice. Adult mice did not develop PE after an intracerebral microinjection of EV71 into the nucleus tractus solitarii (NTS). While viral antigen accumulated in the ventral medulla and the NTS of intracerebrally injected mice, neuronal loss was observed in the ventral medulla only.

**Conclusions:**

Exogenous IL-6, IL-13, and IFN-γ treatment could induce mild PE and exacerbate pulmonary abnormality of EV71-infected mice. However, other factors such as over-activation of the sympathetic nervous system may also be required for the development of classic PE symptoms.

## Background

Enterovirus 71 (EV71), a highly neurotropic, positive-sense single-stranded RNA virus, belongs to the *Enteroviru*s genus of *Picornaviridae *family. In general, EV71 infections are mild, such as hand, foot, and mouth disease and herpangina in young children. However, central nervous system (CNS) infections with life-threatening pulmonary and cardiac complications have occurred [1]. EV71 has been regarded as the most important neurotropic enterovirus since the effective control of the poliovirus (PV). More than a dozen severe EV71 outbreaks have been reported worldwide since it was first recognized in California in 1969 [2].

Pulmonary edema (PE) and subsequent rapid onset cardiopulmonary failure are hallmarks of EV71 induced mortality [3]. EV71-induced PE has been considered neurogenic in origin, as it has been observed to be associated with brainstem encephalitis without signs of pneumonia and myocarditis [4,5]. Most EV71 patients with PE presented symptoms of autonomic nervous system dysregulation and sympathetic excitement, suggesting hemodynamic alterations may underlie the disease mechanism of EV71-induced PE. Elevated levels of plasma catecholamine and epinephrine, coagulative myocytolysis, and myocardial hemorrhage were noted in EV71 patients with brainstem symptoms [6]. Researchers have speculated that systemic and local proinflammatory responses resulting from EV71-related inflammation and brain damage are involved in the development of PE in EV71 patients [7]. However, in one previous study, only 1 out of the 5 PE patients showed systolic hypertension and elevated pulmonary artery pressure [5].

Our previous studies showed that a mouse-adapted EV71 strain, EV71/MP4, could experimentally infect laboratory mice via oral (p.o.), intramuscular, and intracranial (i.c.) inoculation routes, resulting in CNS infection and death [8]. Clinically, the animals developed neurological disease and pulmonary dysfunction. Viral antigens were concentrated in the cerebellar peduncle of the brainstem beneath the cerebellum and the anterior horn regions of the spinal cord, but not in the heart or lungs [9]. The CNS exhibited obvious pathology. However, while the lungs exhibited emphysema, PE was not observed. Our results suggested that CNS infection alone was not sufficient for the development of PE in EV71-infected mice. In this study, we demonstrated that systemic administration of proinflammatory cytokines IL-6, IL-13, and IFN-γ could exacerbate pulmonary abnormalities and induce mild PE in EV71-infected mice.

## Methods

### Cells and viruses

Rhabdomyosarcoma (RD) cells (American Type Culture Collection, Manassas, VA) were maintained in Dulbecco's modified Eagle's medium (DMEM) containing 10% fetal bovine serum, 2 mM L-glutamine, penicillin and streptomycin. Vero cells (ATCC) were maintained in DMEM containing 5% newborn calf serum, penicillin and streptomycin. Mouse-adapted EV71 strain MP4 [8] was propagated in RD cells. Working stocks contained 4 × 10^7 ^PFU/ml. MP4 strains were tested by monoclonal antibodies for EV71 (mAb3324), EV71/coxsackievirus A16 (mAb3323), coxsackievirus A24 (mAb3302), coxsackievirus B3 (mAb3306), echovirus 9 (mAb3313), PV type 1 (mAb3331), PV type 2 (mAb3332), and PV type 3 (mAb3335) (all from Chemicon, Temecula, CA) using indirect immunofluorescence staining of infected RD cell cultures, and RT-PCR using specific primers for EV71. HSV-1/KOS, a clinical isolate strain of human simplex virus-1 (HSV-1) (courtesy of Dr. Shun-Hua Chen, National Cheng Kung University, College of Medicine, Tainan, Taiwan) was grown in Vero cells.

### Animal experiments

Specific-pathogen-free, 7-day-old ICR mice (Laboratory Animal Center, National Cheng Kung University, College of Medicine) were i.c. injected with 10 μl of EV71/MP4 (4 × 10^5 ^PUF/mouse) or HSV-1/KOS (2 × 10^2 ^PFU/mouse) through the fontanelles using a 26-gauge needle. Control mice were given culture medium. To systemically increase proinflammatory cytokine levels, mice were intraperitoneally (i.p.) injected at three days post-inoculation with recombinant mouse IL-6, IL-13, and IFN-γ (0.05, 0.15, and 0.8 μg/mouse, respectively) (R&D, Minneapolis, MN) 2 or 3 times within 48 h. Control mice were given bovine serum albumin (BSA). Mice were observed twice daily for clinical signs and mortality. Lung function and body weight monitored daily for one week. Lung weight/body weight analysis, histopathology, and immunohistochemistry were performed after these experiments. For i.c. microinjection, EV71/MP4 was bilaterally microinjected into the nucleus tractus solitarii (NTS; AP-7.72 mm, LM+/-0.6 mm, DV-5.5 mm) of 8-week-old ICR mice in a volume of 0.2 μl (3 × 10^7 ^PFU/ml) over 10 min through a 27-gauge stainless steel cannula connected to a 10-μl Hamilton syringe (Hamilton, Reno, NV) driven by an injection pump (Legato 200 series, KD Scientific, MA). Mice were observed twice daily for clinical signs and mortality for 5 days. Clinical disease was scored as follows: 0, healthy; 1, ruffled fur and hunchbacked appearance; 2, wasting; 3, limb weakness; 4, limb paralysis; 5, moribund and death. The Institutional Animal Care and Use Committee approved all animal protocols.

### Unrestrained whole-body plethysmograph

Pulmonary functions of EV71- and HSV-1-infected mice were measured in unrestrained animals using a whole-body plethysmograph (Buxco, Troy, NY). Readings were collected for 3 min after 3 min of resting in the chamber.

### Cytokine and noradrenaline levels

After anesthetization with pentobarbital sodium (Nembutal; Abbott Laboratories, North Chicago, IL), blood was collected after axilla dissection. The levels of IL-6, IL-10, IL-13, and IFN-γ in serum and noradrenaline in plasma of EV71-infected mice were determined using ELISA kits (ELISA DuoSet, R&D, Minneapolis, MN) and a noradrenaline EIA kit (LDN, Nordhorn, Germany), respectively according to the manufacturer's instructions. The detection limits for IL-6, IL-10, IL-13, IFN-γ, and noradrenaline were 15.6, 31.25, 62.5, 31.25, and 0.027 pg/ml, respectively.

### Measurement of wet lung weight

Changes in vascular permeability in lungs were determined by measuring the increment of wet lung weight. Individual lung lobes were removed, blot-dried on gauze, and weighed using a balance with accuracy to 0.0001 gram. Data were expressed as the ratio of total wet lung weight to body weight.

### Tissue handling

Animals were perfused with isotonic saline containing EDTA. The whole brains and lungs were removed and weighed. Half of the tissues were immersion fixed in 10% buffered formalin for 48 h, bisected, embedded in paraffin, and stained with hematoyxlin and eosin (H & E) or Nissl stain. The remaining tissue samples were frozen in a liquid nitrogen-cold hexane bath in 100% OCT compound (Miles, Elkhart, IN). All samples were stored at -70° C until assayed.

### Immunostaining

The presence of EV71 virions in cryosections of frozen tissues (8 to 10 μm; Leica CM1800, Wetzlar, Germany) was visualized by a previously described method [8]. In addition, neuronal cells were identified by staining with monoclonal anti-NeuN antibody (Sigma-Aldrich, St. Louis, MO) as previously described [10]. Positively stained cells were enumerated by counting at least 10 fields and expressed as the average number per field.

### Statistical analysis

The clinical scores, lung weight/body weight ratio, and numbers of EV71-positive and NeuN-positive cells were analyzed using either the nonparametric one-way analysis of variance (ANOVA) or Mann-Whitney *U *tests. The results are expressed as means ± standard errors of the means (S.E.M.). A *P *value of < 0.05 was considered significant.

## Results

### EV71 infection provoked proinflammatory cytokine but not noradrenaline production in mice

**Figure 1:**
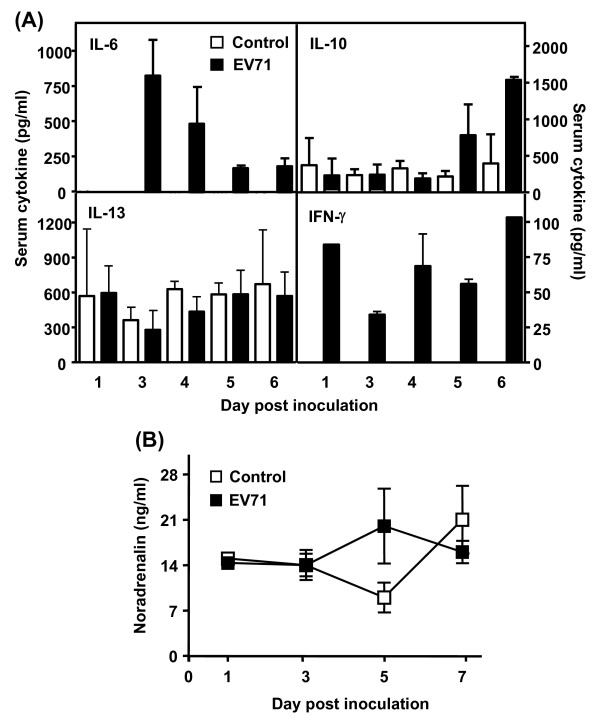
**Intracranial inoculation of EV71 transiently increased proinflammatory cytokines in mice, but not noradreanline**. Seven-day-old ICR mice (n = 24) were intracranially inoculated with or without EV71/MP4 strain (4 x 10^5 ^PFU/mouse). Serum or plasma samples were collected at indicated post-inoculation time points. Concentrations of IL-6, IL-10, IL-13, and IFN-γ (A) and noradrenaline (B) were determined. Data represent means ± S.E.M.

Previous findings suggested that proinflammatory cytokines including IL-6, IL-10, IL-13, and IFN-γ were associated with the development of PE in patients infected with EV71 [7], we thus measured these cytokines in an mouse model of EV71 infection developed by in our laboratory [8]. As expected, after an i.c. inoculation of EV71 (4 × 10^5 ^PFU/mouse), 7-day-old mice did not gain weight and developed clinical symptoms with diffuse emphysema but no evidence of PE in the lung (see Additional File 1, Figure S1). In general, most of the animals died at day 5 post inoculation. There was a significant increase in serum proinflammatory cytokines including IL-6, IL-10, and IFN-γ but not IL-13 (Figure 1A). Specifically, IFN-γ was detected in serum as early as 1 day post infection and remained elevated at a high level for up to 6 days in the survivors. Increased levels of IL-6 were noted 3 days post infection. Elevated IL-10 was only detected at and after 5 days post infection. Notably, there was no increase in the concentration of IL-13 in the serum after EV71 infection. Plasma noradrenaline levels of EV71-infected mice were not statistically difference than those of control mice (Figure 1B).

### Cytokine treatment slightly exacerbated the clinical disease and pulmonary dysfunction of EV71-infected mice

**Figure 2:**
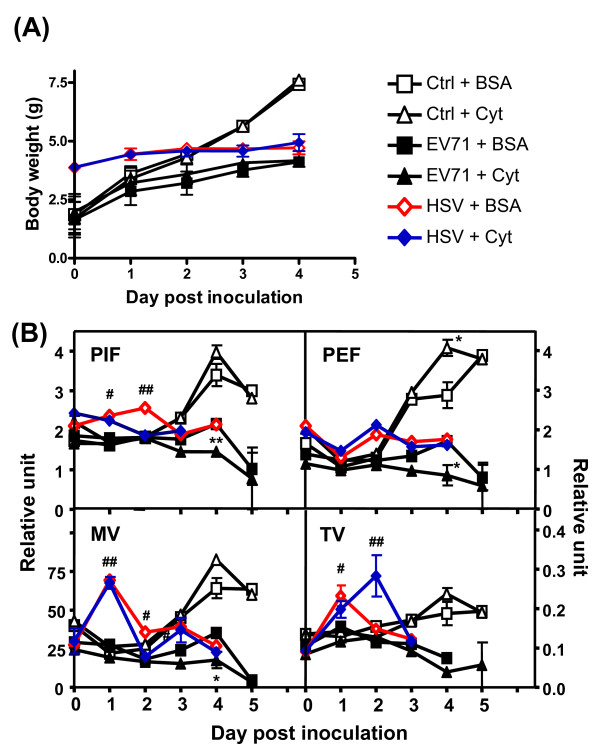
**Proinflammatory cytokine treatment exacerbated pulmonary dysfunction in EV71-infected, but not HSV-1-infected mice**. Seven day-old ICR mice (n = 12) were intracranially inoculated with EV71 (4 × 10^5 ^PFU/mouse) or HSV-1/KOS (2 × 10^2 ^PFU/mouse) followed by an intraperitoneal injection of IL-6, IL-13, and IFN-γ at days 3 and 4 post inoculation (arrows). The body weights (A) and pulmonary functions of mice were monitored daily (B). Ctrl: culture medium; BSA: bovine serum albumin; Cyt: IL-6, IL-13, and IFN-γ treatment; PIF: peak of inspiratory flow; PEF: peak of expiratory flow; MV: minute volume; TV: tidal volume. Data represent means ± S.E.M. *, *P *< 0.05 and **, *P *< 0.0001 as compared to BSA-treatment control. #, *P *< 0.01 and ##, *P *< 0.001 as compared to EV71-infected mice.

To assess whether an insufficient amount of proinflammatory cytokines accounted for the absence of PE in EV71-infected mice, we i.p. injected recombinant mouse IL-6 (0.05 μg/mouse), IL-13 (0.15 μg/mouse), and IFN-γ (0.8 μg/mouse) to mice 3 days after an i.c. inoculation of EV71 (two doses within 48 h). At this time the animals developed mild clinical symptoms with the presence of virus in the CNS. All the mock-infected BSA-treated mice gained weight normally (Figure 2A) without pulmonary dysfunction (Figure 2B). Exogenous IL-6, IL-13, and IFN-γ treatments neither affected the body weight, nor caused illness of the mock-infected mice. As expected, the EV71-infected mice exhibited weight loss, but weight loss was not affected by the cytokine treatments (Figure 2A). Although severe limb weakness and paralysis were observed in some of the infected mice at days 3 and 4 after infection, there was no significant difference in clinical score between the EV71-infected BSA-treated mice and EV71-infected cytokine-treated mice. Conversely, cytokine treatment exacerbated the pulmonary dysfunction of EV71-infected mice as compared with EV71-infected BSA-treated mice at day 4 post inoculation (Figure 2B).

### Cytokine treatment elevated lung weight and induced mild pulmonary edema in EV71- but not herpes simplex virus-infected mice

**Figure 3:**
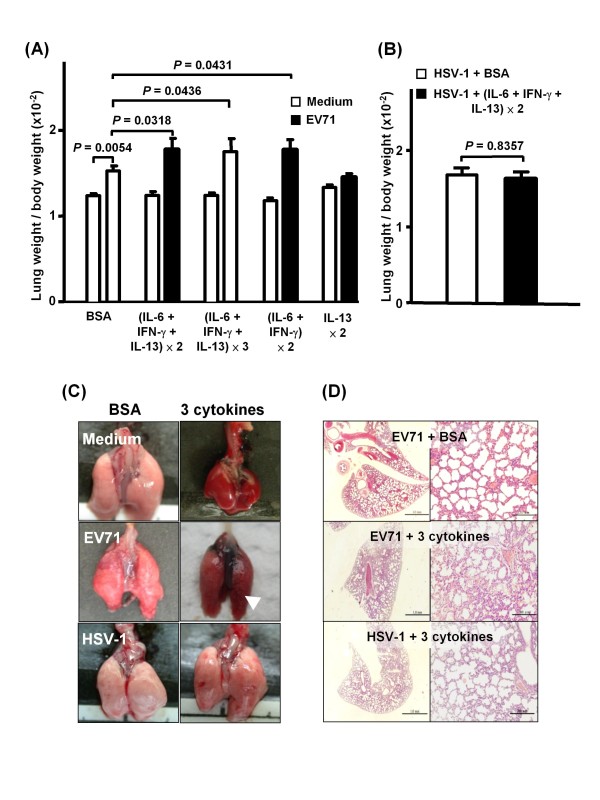
**Proinflammatory cytokine treatment increased the lung weight/body weight ratio and induced alveolar exudation of EV71-infected, but not HSV-1-infected mice**. Seven-day-old ICR mice (n = 12) as treated as described in Figure 2. Their lung weight/body weight ratio was determined (A and B), and lung tissues were collected for gross (C) and histopathological examination (H & E stain) (D) at day 1 after the cytokine treatment. BSA: mice received BSA only; ×2: mice received 2 doses of cytokines within 48 h; ×3: mice received 3 doses of cytokines within 48 h. Note interlobular edema (arrow) (C) and eosinophilic, proteinaceous exudation in alveolar spaces with moderate to severe emphysema in EV71-infected mice with cytokine treatment. Bars: 1.0 mm (left panel), 200 μm (right panel). (D). Data represent means ± S.E.M.

Weight loss in EV71-infected mice resulted in increased lung weight/body weight ratios, as compared with non-infected control mice (*P *= 0.0054, Figure 3A). Exogenous cytokine treatment (IL-6, IL-13, and IFN-γ, 2 doses within 48 h) increased the lung weight/body weight ratio of EV71-infected mice as compared with EV71-infected control mice (*P *= 0.0318, Figure 3A). Diffuse congestion and interlobular edema were noted grossly (Figure 3C). Histopathologically, EV71-infected mice with cytokine treatment exhibited moderate to severe emphysema (>75% alveoli), lung congestion, and mild PE as characterized by the accumulation of eosinophilic and homogenous exudates in alveolar spaces and bronchioles (approximately 12 per 100 alveoli) (Figure 3D). Increasing the dosages of the three cytokines (3 times within 48 h) or IL-13 alone exacerbated the pulmonary dysfunction (see Additional File 2, Figure S2), but did not further increase the lung weight/body weight ratio of the EV71-infected mice (Figure 3A). Co-administration of IL-6 and IFN-γ increased lung weight/body weight ratio of infected mice in a way similar to that of the three cytokines treatment (Figure 3A). To clarify whether the observed effects were specific for EV71 infection, mice were i.c. infected with HSV-1/KOS (2 × 10^2 ^PFU/mouse) prior to the administration of IL-6, IL-13, and IFN-γ. HSV-1 is a neurotropic virus and its infection is known to cause diffuse encephalitis in mice, mainly in tissues of the cerebrum and cerebellu [11]. HSV-1-infected animals developed pulmonary dysfunction with an increase in PIF, MV, and TV as compared to EV71-infected mice before cytokine treatment (Figure 2B). HSV-1-infected mice also exhibited lung emphysema (Figure 3D) with much milder symptoms than those of EV71-infected mice. Exogenous IL-6, IL-13, and IFN-γ treatment neither increased the lung weight/body weight ratio (Figure 3B), exacerbated pulmonary dysfunction (Figure 2B), nor induced PE (Figure 3C and 3D) in HSV-1-infected mice.

### EV71 caused neuronal damage in brain regions not associated with the development of pulmonary edema

**Figure 4:**
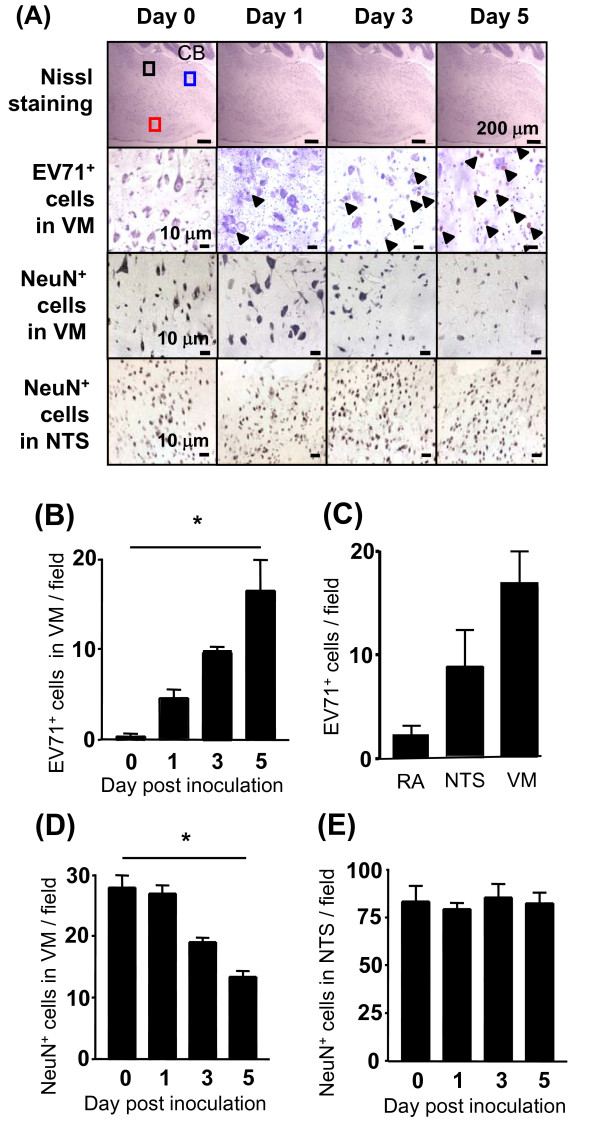
**Microinjection of EV71 to nucleus tractus solitarii resulted in the accumulation of viral antigens in both nucleus tractus solitarii and ventral medulla with neuronal loss in ventral medulla**. EV71/MP4 (3 × 10^7 ^PFU/ml) was bilaterally microinjected into the nucleus tractus solitarii (NTS) of 8-week-old ICR mice in a volume of 0.2 μl. Sagittal cryosections of brain tissues were stained with Nissl strain or anti-EV71 or anti-NeuN antibody (A). Quantification of EV71-positive cells in ventral medulla (VM) (B) and different brain regions (C), and NeuN-positive cells in VM (D) and NTS (E). CB: cerebellum; black square: remote area; red square: VM area; blue square: NTS. Data represent means ± S.E.M. of 3 mice with 3 sections per mouse. *, *P *< 0.001.

To test whether EV71 infection of the NTS was a prerequisite for PE development, we directly i.c. inoculated EV71 into the NTS of adult mice. The animals developed neither clinical disease nor PE over the course of the experiment (data not shown). Immunostaining showed that EV71-positive cells accumulated at the ventral medulla (VM) over time (Figure 4A and 4B). This observation positively correlated with a decrease in the numbers of NeuN-positive cells in this area (Figure 4A and 4D). EV71-positive cells were also detected at the NTS with very few staining outside these areas at day 5 after inoculation (Figure 4C). Surprisingly, the numbers of NeuN-positive cells in the NTS remained unchanged after EV71 inoculation (Figure 4E). These results indicated that EV71 established a lytic infection in the VM and a non-lytic infection in the NTS.

## Discussion

In the course of the development of a mouse model of human EV71 infection, we demonstrated that EV71 could spread from the gastrointestinal tract or muscle to and accumulate in the CNS, especially in the brainstem of mice after different routes of inoculation [9]. Following neuroinvasion, the animals exhibited paralysis, pulmonary dysfunction, and death, but did not develop PE, the hallmark in human EV71 patients with severe complications [12]. In this study, we demonstrated that EV71-infected mice displayed a cytokine profile that was distinct from that of human patients. The NTS was not the CNS target of EV71 infection as viral titer was extremely low in this area of infected mice, which might explain why PE was absent in the infected animals.

Clinical findings showed that increases in serum IL-1β, IL-6, IL-10, IL-13, TNF-α, and IFN-γ were associated with PE development in EV71 patients [7,13,14]. In this study, the EV71-infected mice exhibited only a subtle and transient elevation of serum IL-6, IL-10, and IFN-γ. Thus, the absence of PE in EV71-infected mice might be attributed to inadequate proinflammatory cytokine levels of IL-6, IL-13, and IFN-γ. Indeed, the introduction of exogenous IL-6, IL-13, and IFN-γ in EV71-infected mice exacerbated pulmonary dysfunction and resulted in increases in lung weight/body weight ratio and intra-alveolar exudation. In addition, treatment with IL-6 and IFN-γ was sufficient for provoking the observed effect. Both IL-6 and IFN-γ are related to PE development via their effects on endothelial cells and inflammation [15], [16], and both of these cytokines were important for microvascular angiogenesis and leakage in an atopic dermatitis mouse model [17]. Recent animal study indeed demonstrated the pathological role of IL-6 in the induction of tissue damage and mortality in EV71-infected mice [18]. IL-13 alone exacerbated only pulmonary dysfunction in our EV71-infected mice, which is consistent with the biological function of the cytokine. IL-13 is a potent effector cytokine for asthma, as it can directly cause airway hyperreactivity, mucus over-production, increased pulmonary vessel permeability, and smooth muscle hypertrophy, effects that have been shown to contribute to pulmonary dysfunction and respiratory distress [19]. Taken collectively, our data suggest that elevated serum IL-6 and IFN-γ contribute significantly to PE development during EV71 infection in mice.

Based on the distribution of viral antigens and viral genomic sequences, EV71 is likely propagated throughout the CNS via motor pathways [20]. Our previous study indicated a retrograde axonal transport of the virus in neuronal cells [9], which serves as strong evidence that EV71 preferentially infects certain tissues or cell types in the CNS. Both histopathological [20] and magnetic resonance imaging studies [21], [22] of EV71 patients with PE showed that the major CNS lesions were in the posterior medulla oblongata, pons, midbrain, dentate nuclei of the cerebellum, and ventral horns of the cervical spinal cord. In addition, EV71 viral antigens and genomic sequences were detected primarily in neurons and neuronal processes under inflammatory conditions [23]. The fact that exogenous IL-6, IL-13, and IFN-γ worked only in EV71-infected, but not HSV-1-infected mice, suggests a prerequisite for a region-specific CNS infection for PE development.

Surprisingly, PE did not developed in adult mice after a direct inoculation of EV71 to the NTS, an area known to contribute to the development of PE in rats upon injury [24,25]. In our current study, the neurons in the NTS of adult mice seemed to be more resistant to EV71 than those in the ventral medulla. Thus, it is possible that the inability of EV71 to cause PE was due to the lack of NTS damage and perhaps neurogenic cardiopulmonary complications.

Besides an over-stimulated proinflammatory response, sympathetic excitement has been proposed as another mechanism involved in the development PE in EV71 patients [7]. Imbalances in sympathetic functions have been observed to cause systemic or pulmonary hypertension which may contribute to the development of PE due to hemodynamic alterations and hydrostatic overpressure in mice [26], other animals [26,27], and human beings [28,29]. Moreover, neurogenic stimulation may also induce movement of plasma proteins into the airway lumen in rats [30]. The plasma noradrenaline levels were not altered in EV71-infected mice. Thus, it may worthwhile to test the effects of catecholamine solely or in combination with the cytokines with regard to PE development.

Collectively, we demonstrated that IL-6, IL-13, and IFN-γ over-stimulation exacerbated pulmonary dysfunction and clinical symptoms, and provoked a mild PE in EV71-infected mice. A synergistic proinflammatory cytokine response and damage to specific brain regions, or more precisely specific neuronal cells, may be necessary for the development of EV71-induced PE.

## List of abbreviations

BSA: bovine serum albumin; EV71: Enterovirus 71; HSV-1: herpes simplex virus-1; PIF: peak of inspiratory flow; PEF: peak of expiratory flow; LC: Locus coeruleus; MRI: magnetic resonance imaging; MV: minute volume; NTS: nucleus tractus solitarii; PV: poliovirus; PE: pulmonary edema; TV: tidal volume.

## Competing interests

The authors declare that they have no competing interests.

## Authors' contributions

SWH participated in the design of the study, carried out the animal studies, performed the statistical analysis, and drafted the manuscript. YPL participated in the design of the study. YTH carried out the immunostaining studies. CHL carried out the intracerebral microinjection studies. JIC participated in design and coordinate the immunostaining studies. HYL participated in the design of the study. IJS participated in the design of the study. CKY conceived of the study, and participated in its design and coordination and helped to draft the manuscript. All authors read and approved the final manuscript.

## Supplementary Material

Additional file 1 **Figure 1S. Intracranial inoculation of EV71 resulted in CNS infection, clinical disease, and emphysema in mice**. Seven-day-old ICR mice (n = 24) were intracranially inoculated with or without EV71/MP4 strain (4 x10^5 ^PUF/mouse). Body weight (A) and clinical disease (B) were then monitored daily after infection. Lung tissues were collected for general morphological analysis (H & E stain) (200 x, C; 400 x, D). Clinical disease was scored as followed: 0, healthy; 1, ruffled hair, hunchbacked or reduced mobility. 2, wasting; 3, limb weakness. 4, limb paralysis. 5, death. Bars: 1.0 mm. Note moderate emphysema but not pulmonary edema was observed in the EV71-infected mice (arrowheads). Click here for file

Additional file 2 **Figure 2S. Exogenous IL-13 treatment exacerbated pulmonary dysfunction in EV71-infected mice**. Seven day-old ICR mice (n = 12) were intracranially inoculated with EV71 (4 × 10^5 ^PFU/mouse) followed by an intraperitoneal injection IL-13 at days 3 and 4 post inoculation. Change in pulmonary functions of mice were monitored daily. Ctrl: culture medium; BSA: bovine serum albumin; PIF: peak of inspiratory flow; PEF: peak of expiratory flow; MV: minute volume; TV: tidal volume. Data represent means ± S.E.M. *, *P *< 0.05 and **, *P *< 0.01. Click here for file
