# Supplementary material for: Exogenous interleukin-6, interleukin-13, and interferon-gamma provoke pulmonary abnormality with mild edema in enterovirus 71-infected mice
Source: Respir Res. 2011 Nov 6;12(1):147. doi: 10.1186/1465-9921-12-147 (PMC3223501; doi:10.1186/1465-9921-12-147)
Supplement: Additional file 1 — Figure 1S. Intracranial inoculation of EV71 resulted in CNS infection, clinical disease, and emphysema in mice. Seven-day-old ICR mice (n = 24) were intracranially inoculated with or without EV71/MP4 strain (4 x10^5^ PUF/mouse). Body weight (A) and clinical disease (B) were then monitored daily after infection. Lung tissues were collected for general morphological analysis (H & E stain) (200 x, C; 400 x, D). Clinical disease was scored as followed: 0, healthy; 1, ruffled hair, hunchbacked or reduced mobility. 2, wasting; 3, limb weakness. 4, limb paralysis. 5, death. Bars: 1.0 mm. Note moderate emphysema but not pulmonary edema was observed in the EV71-infected mice (arrowheads). [file 1465-9921-12-147-S1.PPT]

## Slide 1
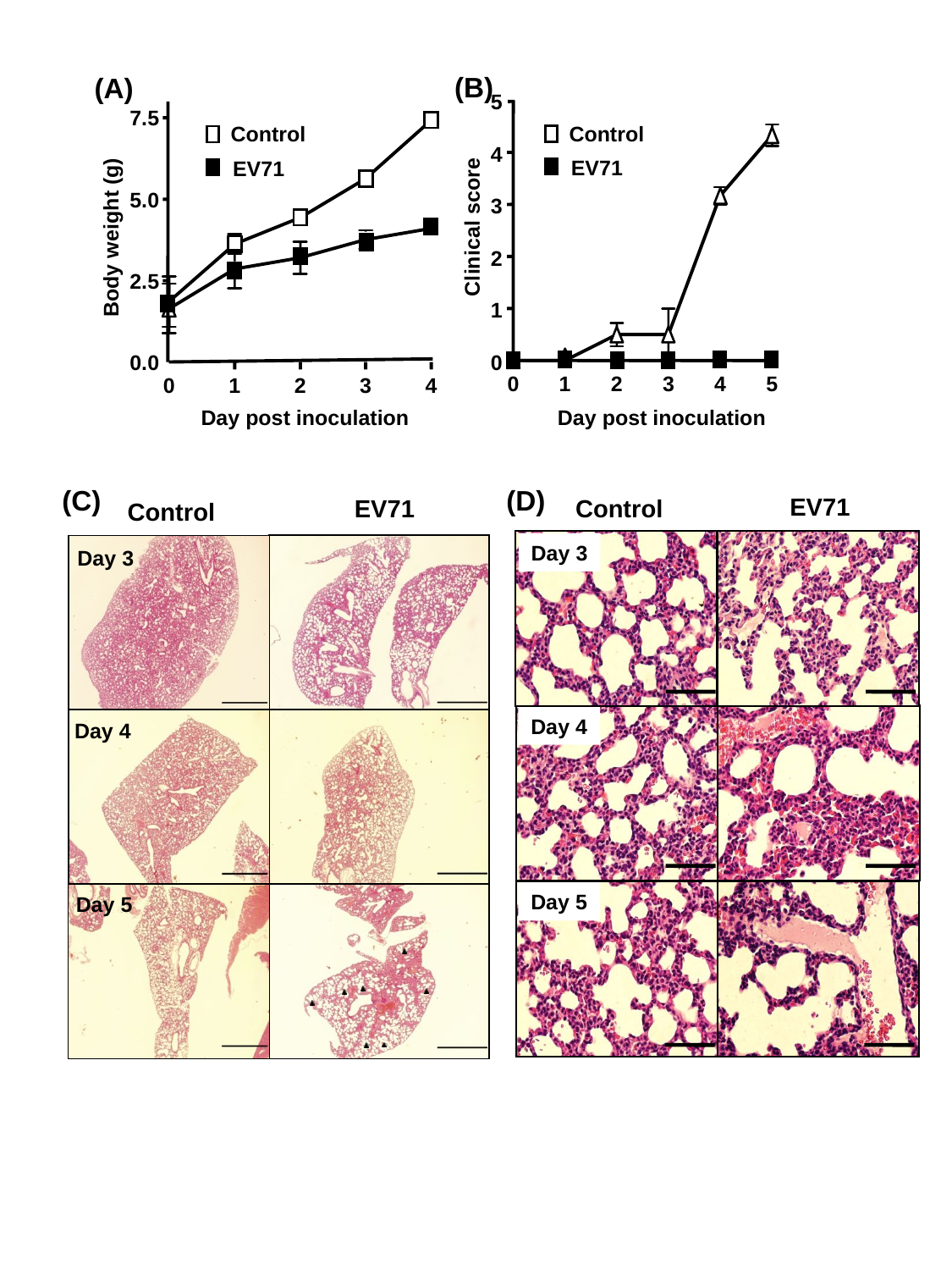

(B)
(A)
7.5
5.0
Body weight (g)
2.5
0.0
0
1
2
3
4
Day post inoculation
5
 Control
EV71
 Control
EV71
4
3
Clinical score
2
1
0
0
1
2
3
4
5
Day post inoculation
(C)
EV71
Control
Day 3
Day 4
Day 5
(D)
EV71
Control
Day 4
Day 5
Day 3
