# Supplementary figures and images for: Exogenous interleukin-6, interleukin-13, and interferon-gamma provoke pulmonary abnormality with mild edema in enterovirus 71-infected mice
Source: Respir Res. 2011 Nov 6;12(1):147. doi: 10.1186/1465-9921-12-147 (PMC3223501; doi:10.1186/1465-9921-12-147)

## Slide 1
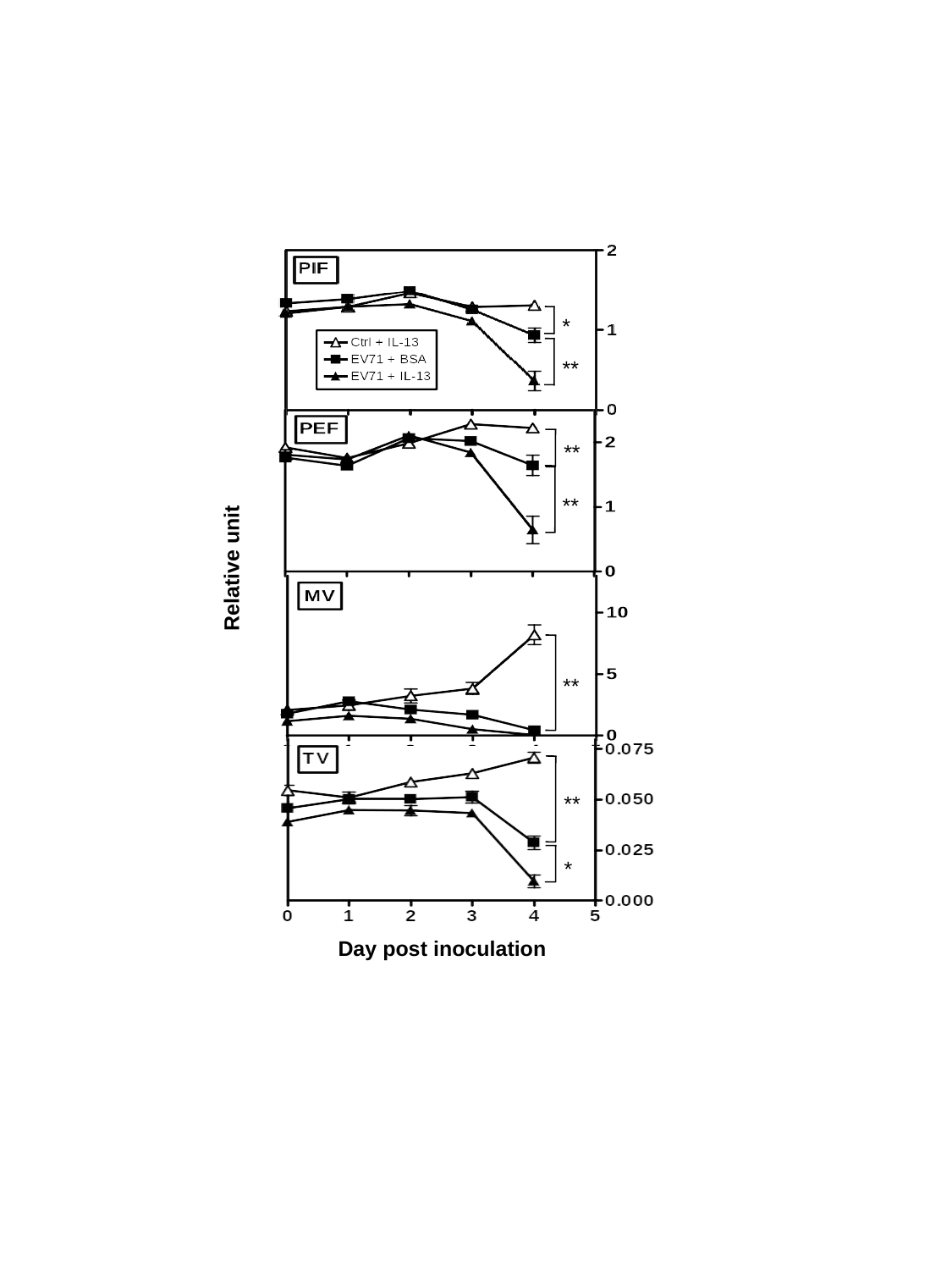

*
**
**
**
Relative unit
**
**
*
Day post inoculation

Supplement: Additional file 2 — Figure 2S. Exogenous IL-13 treatment exacerbated pulmonary dysfunction in EV71-infected mice. Seven day-old ICR mice (n = 12) were intracranially inoculated with EV71 (4 × 10^5^ PFU/mouse) followed by an intraperitoneal injection IL-13 at days 3 and 4 post inoculation. Change in pulmonary functions of mice were monitored daily. Ctrl: culture medium; BSA: bovine serum albumin; PIF: peak of inspiratory flow; PEF: peak of expiratory flow; MV: minute volume; TV: tidal volume. Data represent means ± S.E.M. *, P < 0.05 and **, P < 0.01. [file 1465-9921-12-147-S2.PPT]
